# Probable Appearance, Cause and Prevention of Cholera

**Published:** 1867-06

**Authors:** 


### Editorial Department.

### Probable Appearance, Cause and Prevention of Cholera.

The approach of the warm season renews the old and anxious inquiries concerning cholera, that scourge and dread of large cities. Shall we have cholera this season? Is it contagious? Can it be prevented in its appearance by quarantine or municipal regulations? These are the most common and important inquiries concerning it, questions which have been proposed to the most observing and experienced in our profession, as well as to all others, regardless of the possibility of satisfactory answer.

Last year Buffalo was remarkably exempt from epidemic cholera, but this does not justify the conclusion that the warm season, now commencing, will not furnish cases of this disease. It is well to consider the liability, and to adopt such measures of protection as have been found most efficacious. Whatever differences of opinion may prevail in regard to the causes and modes of propagation of cholera, some facts are already established, and upon these may rest a sanitary and municipal action, which will protect the inhabitants in great degree, at least, from the ravages of this fearful scourge. The filthy, vicious, intemperate, destitute and imprudent, fall ready victims to this disease, and from this fact alone may be gathered some of the most important practical lessons; yet the public are as insensible and stupid as possible as to the importance of the various hygienic methods of preventing disease of this nature. The wealthy console themselves with the idea of seeking personal safety in country or sea-side resorts, and bestow little thought upon the value of general health, to city or town, other than possibly to reckon upon its commercial bearings and draw a balance between the taxable cost of sanitary measures and the undertaker's bills for burying the poor. If there was a just appreciation of the injurious effects of filthiness, particularly in yards, sinks, privies, and cesspools; of the danger of stagnant water under and around thickly inhabited districts; of the necessity of drainage and ventilation, there would not be found in Buffalo one nuisance of this sort where hundreds at present exist. As it now is, many families with ample incomes live in dwellings wholly unsuited for the abodes of men, and upon all sides are surrounded by intolerable nuisances. The wonder is, not that we have disease in the warm season, as the result of such conditions, but rather that any can live to reasonable age under such influences. The amount of preventable disease and the number of preventable deaths, if estimated truthfully would astonish every one—there can be no doubt they would outnumber those from unavoidable causes.

Is cholera contagious? This question has not been answered by unanimous opinion, and at present cannot be determined beyond controversy. Is its cause a material generated by morbid processes in the bodies of the sick, and capable of generating the same disease in those whom it reaches, either by contact or through the air? Some of the reasons for believing that in this sense it is not contagious, are the following: Diarrhœa is generally observed to precede and accompany this disease, which in some cases from local or other causes increases and becomes cholera, while in others it remains simple diarrhœa, showing a more general influence than could be expected if the disease was contagious and depending upon it alone as means of propagation. Again, its sudden disappearance, with no reference to the number of persons exposed, or liable as ever to exposure, appears opposed to the idea of contagion. The simultaneous appearance of cholera in places widely separated, and the attack of hundreds the same day in the same city, as has been often observed, indicate its causes to be more general than could arise from contact or near connection. Its appearance in limited districts of a town, to an almshouse or jail, generally containing some cesspool or overflowing sink, when other places do not contain even single cases of the disease, indicate that it is not propagated by contagion. That armies sometimes have suffered terribly while in one location, and by a short march escaped it altogether, is also indicative of local or endemic causes rather than otherwise. Nurses, even in hospitals, are not more subject to the disease than others, and hospital patients suffering from other diseases, have not generally been affected by the admission of those sick of cholera. Physicians are often greatly exposed both in the care of cholera patients while living, aud by making *post mortem* examinations, and no ill effects have been shown to follow. Cholera has never been produced experimentally by direct contact or otherwise, inoculation, tasting the dejections, and inhaling the breath have all alike proved unsuccessful. If possible it is better to reject the idea of contagion, for if adopted it is capable of nntold evil. When asked if cholera is contagious, the answer should be, no, without any hesitation, and this reply should be made and repeated until it can be shown clearly to be untrue.

The remaining question, can cholera be prevented by quarantine or municipal regulation, admits of discussion, but may undoubtedly be answered, yes. It may not be possible with our present knowledge to wholly prevent the appearance and spread of cholera, but it can be prevented in great degree. It is dependent upon local causes to a great extent, at least, and may be controlled and prevented by faithful adherence to the well understood principles of hygienic science. These embrace cleanliness, the use of disinfectants, ventilation with proper air-space, the use of pure water only and proper food. Other points may be also added, such as warm clothing, regular hours of labor and rest, temperance, mental composure, cheerfulness, etc., etc.

A great many facts bearing upon all these topics have been carefully observed and recorded during the past year, and an honest and earnest effort has been made to determine by positive proof or accumulated evidence the various questions in agitation as to the nature, causes, modes of propagation and treatment of this, as yet, ungovernable scourge. Many authors have written papers, and even books, upon cholera, which contain valuable suggestions and will prove instructive, but it must be confessed that these efforts have generally been in the interest and support of some favorite theory. Report favorably the facts as they appear and theory will soon conform to them. Observations are sometimes deceptive, and too great readiness to arrive at conclusions should be carefully avoided.

